# O2O selection mode portrait and optimization for railway service enterprises based on *K*-means

**DOI:** 10.1007/s40747-021-00375-0

**Published:** 2021-05-05

**Authors:** Changsuo Sun, Long Ye, Na Zhang

**Affiliations:** School of Economics and Management, Beijing Jiaotong University, Beijing, 100044 China

**Keywords:** Railway service company, O2O, Supply chain, Optimization

## Abstract

Establishing a platform successfully is just the basis for railway service companies to meet the demands of online to offline (O2O) supply chain services. In this paper, the *K*-means algorithm is first used to construct the user segmentation model of railway service companies and the AISAS (Attention-Interest-Search-Action-Share) method is used to establish the evaluation O2O model. According to this result, we propose four modes to establish O2O supply chain service platform for railway enterprise, which are self-built and self-operated (SBSO, Mode1), commissioned construction and self-operated (CCSO, Mode2), self-built and commissioned operation (SBCO, Mode3), commissioned construction and commissioned operation (CCCO, Mode4). By comparing the advantages and disadvantages of the four modes, the results illustrate the optimal model is impacted by the nature of the platform's operating products and the operating capabilities of the partners. The railway service enterprise needs to transform the traditional multi-level management model into the flat model to adapt the O2O supply chain strategies.

## Introduction

With the advent of the “Internet + ” era, making passengers can enjoy timely and efficient railway and supply chain services without leaving home has generated a higher demand, O2O has become one of the most valuable transformations of the railway service enterprises development path. More recently, COVID-19 related travel restrictions have led many services to quickly transition to online [1]. It also accelerates the pace of O2O change for railway service enterprises to adopt O2O strategies, either completely or partially in collaboration with third-party platforms.

The railway supply chain O2O model has already seen the beginning in China. Shandong Jinan Railway Station has accelerated the construction of service system, initially perfected the service system of “one center, two channels and one platform”, and built up an O2O model of passenger service. One center is the customer service center; two channels, one channel is the online microblogging, WeChat and telephone reservation service, the other channel is the offline love channel. Through official microblogging, WeChat groups, friends' circles and other forms, the train station uses the internet to “cross-pollinate” with the station's passenger sections and all the larger stations across the country, and establishes cooperation with long-distance bus, aviation and other transportation departments and other public welfare organizations to realize information sharing and collect information flow on passenger needs. The train station has built the customer service center into a three-dimensional operation platform that integrates the service center, comprehensive center and emergency center, and provides special services, such as key passenger ticket windows, service posts, inbound passageways, customer service centers, passenger ticket-cutting gates, one-key help system on the platform and transit area at the exit gate, through direct-dial telephone, love relay cards and “one-to-one private customized services”. “Open up the connection, to achieve key passenger services” final closed loop. In the railway supply chain O2O model, it can also provide a richer range of services. For example, passengers can also place orders in the app to provide corresponding products at stopping points during the journey. The railway O2O model has great potential for market broadening and is deepening and diversifying towards a multi-level, multi-dimensional complex ecosystem industry.

The establishment of O2O platform is the foundation for railway service enterprises is the first challenge, and then satisfying the needs of passengers by online is another important issue faced by railway service enterprises. The studies will propose four different model to construct platform and analyze their comparative advantages, and then select the most appropriate construction mode. In fact, with the advancement of technology, passengers' time is gradually fragmented and their demand for products/services becomes more and more personalized. When a wide variety of apps are available, railway service companies need to change their organizational structure to seize users' time and smoothly deliver their products/services to the target customers. Furthermore, O2O may prompt railway service companies transform the traditional multi-level management model into a flat model, in which setting up operations departments or outsourcing operations could ensure the normal functioning of the entire O2O supply chain services.

The rest of the paper is organized as follows: We first conduct a comprehensive literature review in Sect. Literature review. In Sect. Selection mode portrait, an analytical portrait model and the mathematical model are proposed that forms the basis of the research problem. In Sect. Modeling, solution and analysis, we compare the four modes and draws corresponding conclusions. In Sect. Analysis of the four modes, we verify the model through numerical simulation. Section Numerical simulation concludes findings and gives practical suggestions.

## Literature review

At present, the O2O model is developing rapidly in the retail, service and other industries, and the railway service industry has just started, and few researchers have paid attention to the development of O2O in railway service enterprises. In this paper, we mainly summarize the existing research progress from the O2O business model and service supply chain.

### O2O and business mode portrait

Rampell argued that the core of the O2O business model is to search for customers online for offline purchases and to combine the payment model with offline store traffic, and draws on e-commerce B2B, B2C and other common naming abbreviations such as O2O [2]. Fang argued that today's O2O has long since ceased to be a separate online one offline concept, and has added more operational methods, but O2O commerce itself is still oriented to the field of life consumption [3].

The development of O2O business model in China originated from the emergence of online group buying model [4], and before 2010, Ctrip.com was the first O2O business model in China, represented by the intermediary model of online solicitation of customers and offline acquisition of travel company services provided to customers [5]. According to Yu [6], community O2O is a mobile Internet-based platform that integrates online and offline resources to provide a “last mile” service system for community residents, while Shen [7] believed that the unique attributes of the property industry give it a natural soil advantage over other traditional industries. Under such a background, the property industry has also set off a wave of community O2O business model change within the industry. More and more property service companies are beginning to rethink the value of travelers and actively participate in community living O2O construction while upgrading and optimizing basic property services to deeply explore and enhance the value of property services [8].

Most scholars are optimistic about the driving effect of O2O model on the existing market. Zhang and Moonargued that the efficient and convenient nature of O2O business model is attractive to both buyers and sellers and is a win–win-win model for merchants, customers, and potential participants [9]. Won et al. pointed out that O2O platforms can meet the diverse needs of buyers and sellers and improve the efficiency of transactions, and mobile marketing is the key to the development of this model [10]. Shenet al. used empirical research methods to explore the differences between the O2O model and the traditional model, that consumers online good word-of-mouth feedback, as well as learning from other successful business models can help strengthen the expansion of online and offline platform business and profitability, but also increase the industry competition [11]. Xu believed that with the rapid development of mobile Internet, the emergence of smartphones, tablets and other mobile terminals has changed people's lives, shopping patterns and consumption habits [12]. Hui also analyzed the future opportunities and challenges of m-commerce by investigating the background and current situation of m-commerce and O2O business models [13]. Xing et al. [14], Wan [15] pointed out that marketing, transaction and consumption experience are the most active basic business in the business model. Behavior, using these 3 behavioral coordinates to define O2O, the following 4 types of business models can be derived: online merchant transactions-offline consumer experience, online shared transactions -offline usage experience (sharing economy model), offline merchant marketing-online transactions to place orders, offline merchant marketing-order online transactions and synchronize online and offline.

### O2O and supply chain optimization

Gao and Su analyzed the impact of “buy online, pick up offline” and developed a programmatic model with online and offline channels based on consumers' channel choice preferences [16]. Products, but purchased online at lower retail prices, studied two types of online and brick-and-mortar retailers and analyzed the price matching problem [17]. Tsay and Agrawal studied the impact of channel conflict on consumer demand in an e-commerce environment and classified channel sales strategies into direct sales, sales through distributors and both combined with sales while developing incentive programs [18]. Ullrich and Transche discussed the relationship between supply-supply mismatches (DSM) and stock returns and performed an empirical analysis, and DSM reduced sales growth rates and profitability [19]. Cao et al. found that manufacturers could using their own retail stores to sell more products to increase market demand, if manufacturers sell highly substitutable products, they should use independent retailers and consider the cost of product returns. Chocarro et al. discussed the market demand and channel selection issues for online and offline retail stores [20]. Balakrishnan et al. analyzed the market demand and channel selection issues for online and offline retail stores in a demand uncertainty scenario, the influence of browsing and switching behavior on online pricing decisions. Jain et al. [21] studied the problem of optimal decision making based on the capabilities of supply chain members and service quality for different products when considering integrated or decentralized situations. After-sales service framework and its application. Resta et al. [22] discussed the issue of optimizing supply chain management by optimizing product design and improving the service quality. Caggiano et al. [23] studied the issue of multi-product, multi-stage supply chain to improve supply chain efficiency by optimizing consumer-facing service quality and inventory. Dan et al. pointed out that although O2O promotes the change of e-commerce, it brings various drawbacks such as unequal information, questionable authenticity of goods, and the quality of service is also not guaranteed [24]. O2O e-commerce model may overturn the original organizational structure, the original single organizational structure, will evolve into multiple divisions or multiple departments coordinated development [25].

Nowadays, the research of O2O supply chain services of railway is still in a blank stage, and the main reason may be the reform hysteresis of state-owned railway services enterprise facing the shock of O2O business model. However, with the maturity of technology and the introduction of relevant policies, it is an inevitable for railway service enterprises to reform. In this case, the study was conducted with an assumption scenario that railway service enterprises are seeking for O2O reform, which faces the strategic decision of building an O2O supply chain service platform either self-built or entrusted to a third party and operating mode self-operated or entrusted to a third party. And it points out possible results for railway services company after reforming.

## Selection mode portrait

### K-means method

We use the vector $V = (V_{1} ,V_{2} , \ldots ,V_{K} )^{T}$ to depict the Euclidean metric for computing the distance between points and cluster centers in K-means algorithm. As a result, K-means can find spherical or ball-shaped clusters by data analysis. Square sum of error is often used as the criterion function for K-means clustering, so we use the definition:

1 $$ E = \sum\limits_{i = 1}^{k} {\sum\limits_{{p \in X_{i} }} {\left\| {\left. {p - m_{i} } \right\|} \right.} }^{2} $$

where $m_{i}$ is the mean, $p$ means the spatial spot.

**Fig. 1:**
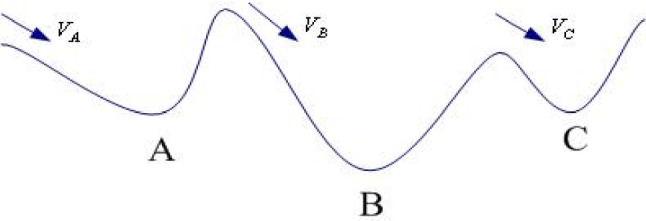
The local minimum and whole minimum

The initial value vectors $$ V_{A} V_{B} V_{C}$$ could be gradually reduced, so the corresponding minimum values could be got, respectively. And among them, the value at point B is the global minimum, the values at point A and C are the local minimum (see Fig. 1).

We set $X=\left\{{x}_{i}\right\},i=1,\ldots ,n$ clustered into a set of *K* clusters, and $C=\left\{{c}_{k},k=1,\ldots ,K\right\}$*.* *K*-means is used to find the value at point B which is the global minimum. ${z}_{j}$ is the mean of cluster ${c}_{k}$, and the squared error between ${z}_{j}$ and the points ${c}_{k}$ is:

2 $$J\left({c}_{k}\right)=\sum_{{x}_{i}\in {c}_{k}}{\Vert {x}_{i}-{z}_{j}\Vert }^{2}$$

K-means is used to minimize the sum of the squared error in all K clusters.

3 $$ J_{C} (I) = \sum\limits_{j = 1}^{k} {\sum\limits_{k = 1}^{{n_{j} }} {\left\| {x_{k}^{(j)} - Z_{j} (I)} \right\|}^{2} } $$

The main steps of K-means algorithm are as follows:

1. Given data set *n*, let *I* = 1, $Z_{j} (I), \, j = 1,2,3, \ldots ,k$ is selected as the initial clustering centers;
2. Calculate the distance between all points and clustering center:
  $$D(x_{i} ,Z_{j} (I)), i = 1,2,3, \ldots ,n, \, j = 1,2,3, \ldots ,k$$,
  If $$D(x_{i} ,Z_{k} (I)) = \min \left\{ {D(x_{i} ,Z_{j} (I)), \, j = 1,2,3, \ldots ,k} \right\}$$, $${\text{then }}x_{i} \in w_{k}$$;
3. Calculate the error function $J_{C}$;
4. If $\left| {J_{C} (I) - J_{C} (I - 1)} \right| \triangleleft \xi$, the algorithm ends; otherwise, the algorithm finds new clustering centers:
  4 $$ Z_{j} (I) = \frac{1}{n}\sum\limits_{i = 1}^{{n_{j} }} {x_{i}^{(j)} , \quad j = 1,2,3, \ldots ,k} $$

**Fig. 2:**
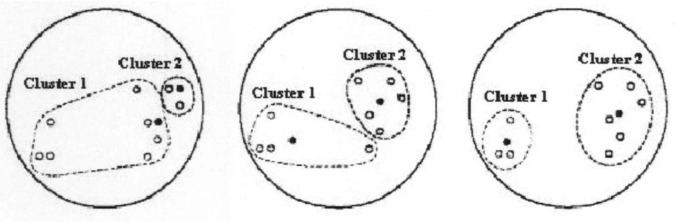
The diagram of K-means algorithm

The iteration of K-means is showed in Fig. 2.

AISAS (Attention-Interest-Search-Action-Share), consumers gradually change from passively receiving product information and marketing propaganda to actively acquiring and cognizing, AISAS emphasizes information collection after consumers pay attention to products and generate interest (AISAS considers information gathering and information sharing as two important aspects, both of which are inseparable from consumers' use of the Internet. The Internet provides consumers with the opportunity to actively access information, so that they can obtain detailed professional information from multiple sources and make relatively “informed” purchases. However, under the AISAS model, the core driver of marketing activities is still advertising, and the keywords of marketing activities are still brand display and impression, but there are more click, action, Landing page conversion and other such effect dimensions, and the relationship between brands and users has started to Although the relationship between brands and users has started to interact, it is only based on the simple fragmented feedback of links, rather than the multi-point two-way system interaction based on connections. The AISAS model is still fundamentally based on advertising to generate Attention, a linear one-way marketing communication process, and a behavioral consumption process. In fact, Share and experience sharing are becoming the source of consumption in the real sense. The parameters of AISAS are depicted in Table 1.

**Table 1:** The parameters of AISAS

Parameters	Implication
TT	The number of offline users serviced by railway service enterprises
FL	The quantity of railway service enterprises
HT	The number of online users serviced by railway service enterprises
*T*	Time point of calculation
*t-1*	Time point of calculation before *t*
HT_*i*_	The number of online users serviced by railway service enterprises during time point *O* to *t*
HT_*i-1*_	The number of online users serviced by railway service enterprises during time point *O* to *t-1*
*△*HT	The number of users in different mode between *t* and *t-1*
$\alpha_{0}$	Railway service enterprises' impact on early O2O user phase
$\alpha_{1}$	The coefficient of *HT*_*i-1*_ which is [0,1]
$\alpha_{2}$	The coefficient of different O2O mode

Given the time point *t* and *t-1*, railway service enterprises' impact on early O2O user phase from *O* to *t* is defined as

5 $${HT}_{i}={S}_{AA/B/D/DBA}$$

and the cumulated impact from *O* to *t* is calculated as

6 $${HT}_{i-1}={S}_{A/B/C/CBA}$$

Therefore, the number of users in different mode between *t* and *t* − 1 is

7 $$\Delta HT={HT}_{i}-{HT}_{i-1}={S}_{C/D/DC}$$

The AISAS clustering centers based on K-means method is

8 $${HT}_{i}={\alpha }_{0}+{\alpha }_{1}\bullet {HT}_{i-1}+f(TT, FL)$$

### Portrait results

In the model, the railway service company users can be abstracted into a topological graph $G(V,E)$, where $V$ represents the set of all nodes (including railway service company and users and alternative points), $E$ represents the set of all arcs, and $G(V,E)$ represents the graph composed of all points and edges through certain connection relations. Next, given the demand of railway service company and the number of each demand: (1) the total number of $\kappa$ such demands, number $k=\mathrm{1, 2}, \dots ,\kappa$, the set of all demands is $\kappa$; (2) the size of the $\kappa$ th demand is ${h}^{k}$, the online and offline are ${o}^{k}$ and ${d}^{k}$, respectively, $O$($D$) denotes the set of online and offline of all demands; (3) ${w}^{k}$ represents the importance of the $\kappa$ th demand. ${l}_{ij}$ denotes the edge between nodes $i$ and $j$, and whether the $\kappa$ th demand passes through. ${l}_{ij}$ is denoted by ${x}_{ij}^{k}$. In addition, the length of each edge in the graph is ${c}_{ij}$ and the maximum number of visits allowed by the edge is ${u}_{ij}$. The number of modes placed at each node is less than *p*. The objective function is to minimize the total cost.

$$min\sum \limits_{k\in K}\sum _{i\in V}\sum _{j\in V}{c}_{ij}{x}_{ij}^{k}{{h}^{k}w}^{k}$$

Subject to

9 $$\sum \limits_{j\epsilon V}{x}_{{o}^{k}j}^{k}=1, \quad \forall k\in K$$

10 $$\sum \limits_{i\epsilon V}{x}_{i{d}^{k}}^{k}=1, \quad \forall k\in K $$

11 $$\sum \limits_{j\epsilon V/O}{x}_{ij}^{k}=\sum _{j\epsilon V/D}{x}_{ji}^{k}\quad \forall i\in V/\{O\cup D\},k\in K$$

12 $$\sum \limits_{k\epsilon K}{x}_{ij}^{k}{h}^{k}\le {u}_{ij},\quad \forall i\in V, j\in V$$

13 $${f(x}_{ij}^{k})\le n \quad \forall j\in V$$

14 $${x}_{ij}^{k}=\left\{\mathrm{0,1}\right\}\quad \forall k\in K,i\in V,j\in V$$

Constraints (9) and (10) denote that each user demand has and has only one online and offline, respectively. Constraint (11) is a user access balance constraint indicating that each user demand has only one online and offline preference. Constraint (12) is the user access constraint for each arc. Constraint (13) is the user access constraint for each mode. The function ${f(x}_{ij}^{k})$ denotes the number of non-repeating endpoints of the access demand through node $j$. Let the set ${D}_{j}$ store all the endpoints of access demands that pass through $j$. First calculate ${x}_{ij}^{k}{d}^{k}$ and store it into the set ${D}_{j}$ if ${x}_{ij}^{k}{d}^{k}$ is not equal to 0. Delete the duplicate items in ${D}_{j}$ to get ${D{^{\prime}}}_{j}$, and the number of elements in ${D{^{\prime}}}_{j}$ is ${f(x}_{ij}^{k})$. Constraint (14) indicates that ${x}_{ij}^{k}$ is a 0–1 variable.

In this paper, the clustering centers were established when *K* = 4, which represent the four clusters. The details were shown in Tables 2 and 3.

**Table 2:** The center of cluster

Mode	O2O expense/10,000	Disposable income/10,000	Personal income/10,000
SBSO	0.68	10	36.5
CCSO	1.61	12.1	28.2
SBCO	3.2	8.6	22
CCCO	4.1	10.3	28

**Table 3:** The distribution of different mode

	SBSO	CCSO	SBCO	CCCO
Number of samples	2860	3507	5032	5344
Rate (%)	17.7	26.8	29.1	26.4

The effect of *K*-means algorithm was evaluated by supervision and non-supervision method, respectively. The results were shown in Table 4.

**Table 4:** The results of cluster

	Inner cluster distance	Cross cluster distance	*V*	Accuracy
*K* means	0.041	0.432	0.079	88.4%

The portrait results were shown in Table 5.

**Table 5:** The portrait of different mode

O2O mode	Mode and user number	*R*^*2*^	AISAS parameter	Association coefficient	*T *Statistic	Probability
SBSO	0.992	0.989	FL	0.015	2.689	0.011
			HT_*i-1*_	0.832	15.769	0.000
			${\alpha }_{0}$	13.167	2.101	0.042
CCSO	0.945	0.998	FL	0.280	4.899	0.000
			HT_*i-1*_	0.874	35.309	0.000
			${\alpha }_{0}$	− 813.002	− 3.619	0.001
SBCO	0.971	0.999	FL	0.210	1.531	0.134
			HT_*i-1*_	0.916	19.761	0.000
			${\alpha }_{0}$	− 2279.52	− 1.431	0.159
CCCO	0.995	0.998	FL	− 0.213	− 2.481	0.016
			HT_*i-1*_	1.048	115.441	0.000
			${\alpha }_{0}$	− 19.138	− 0.791	0.433

Obviously, due to the lack of experience and foundation of self-build/self-operate, the companies tend to bring weaker results with the same effort as third-party companies. Based on the above background, the study assumes that there is a railway service company in the market that wants to change its O2O supply chain service, and it is faced with the decision of building an O2O supply chain service platform (self-built or entrusted with a third party) and operating model (self-operated or entrusted with a third party). As a result, the enterprise is faced with four choices: self-build and self-operate, commission build and self-operate, self-build and commission operate, and commission build and commission operate.

## Modeling, solution and analysis

### SBSO, mode1

When the railway service company (A) chooses to build its own supply chain online platform and operate itself, it needs to decide the degree ($\theta$) of effort to build the platform, and the corresponding cost is $k_{1} \theta^{2} {/2}$, at this time, because the platform only has the ability to integrate merchants and customers, and display products/services Such basic functions cannot bring more drainage effects. Therefore, the customer group coverage under the self-built mode is ($\theta$); because the enterprise chooses to operate by itself, the enterprise needs to decide the degree of self-operation effort $e$, and the corresponding cost is $k_{2} \theta^{2} {/2}$, the drainage effect is $\alpha e$, and now the profit function of the enterprise can be expressed as:

$$ \begin{array}{*{20}c} {\max } & {\pi_{A} } \\ \end{array} (\theta ,e) = (\theta + \alpha e)p - \frac{{k_{1} \theta^{2} }}{{2}} - \frac{{k_{2} e^{2} }}{{2}} $$

In the above formula, the total revenue of the first term ($\theta + \alpha e$) is the number of customers,$p$ is the revenue per customer), the second term is the cost of platform establishment, and the third one is the operating cost. To maximize $\pi_{A} (\theta ,e)$, let $\pi_{A} (\theta ,e)$ = 0 to get its partial derivation.

$$ \left\{ {\begin{array}{*{20}c} {\frac{{\partial \pi_{A} (\theta ,e)}}{\partial \theta } = p - k_{1} \theta = 0} \\ {\frac{{\partial \pi_{A} (\theta ,e)}}{\partial e} = \alpha p - k_{2} e = 0} \\ \end{array} } \right. $$

The result is

$$ \left\{ {\begin{array}{*{20}c} {\theta^{*} = \frac{p}{{k_{1} }}} \\ {e^{*} = \frac{\alpha p}{{k_{2} }}} \\ \end{array} } \right. $$

Put $(\theta^{*} ,e^{*} )$ into $\pi_{A} (\theta ,e)$, we get

$$ \pi_{A} (\theta^{*} ,e^{*} ) = \frac{{p^{2} }}{{2k_{1} }} + \frac{{\alpha^{2} p^{2} }}{{2k_{2} }} $$

### CCSO, mode 2

When a railway service company chooses to entrust a third-party company (B) to build a supply chain online platform and operate it itself, just like the model, A needs to decide the degree of self-operation effort $$e$$, Since the construction of the supply chain online platform is entrusted to a third-party company, A needs to make a decision to pay B's platform construction costs $$w_{1}$$($$\underline{{w_{1} }} \le w_{1} < \overline{{w_{1} }}$$), and B needs the effort of making a decision-making platform $$\theta$$ (A stipulates that the effect of company B's construction platform should be no worse than the effect of A's self-built, that is $$\theta \ge p/(1 + \eta )k$$, and the costs accordingly is $${\raise0.5ex\hbox{$\scriptstyle {k_{{3}} \theta^{2} }$} \kern-0.1em/\kern-0.15em \lower0.25ex\hbox{$\scriptstyle 2$}}$$($$k_{3} < k_{1}$$). At this time, because B has the corresponding foundation and can bring more drainage effects, the customer group coverage under the entrusted third-party enterprise construction model $$(1 + \eta )\theta$$, therefore, the profit function of an enterprise can be expressed as:

$$ \begin{gathered} \begin{array}{*{20}c} {\max } & {\mathop {\pi_{A} }\limits_{{\underline{{w_{1} }} \le w_{1} < \overline{{w_{1} }} }} } \\ \end{array} (w_{1} ,e) = [(1 + \eta )\theta + \alpha e]p - w_{1} - \frac{{k_{2} e^{2} }}{{2}} \hfill \\ \begin{array}{*{20}l} {s.t.} \hfill & {\mathop {\pi_{B} }\limits_{{\theta \, \ge \, \frac{p}{{(1 + \eta )k_{1} }}}} (\theta ) = w_{1} - \frac{{k_{3} \theta^{2} }}{{2}}} \hfill \\ {} \hfill & {\pi_{B} (\theta^{*} ) \ge 0} \hfill \\ \end{array} \hfill \\ \end{gathered} $$

In the objective function above, the first item is total revenue, the second item is the transfer payment for entrusting a third-party enterprise to build the platform, and the third item is the operating cost. Enterprise A needs to decide the size of the transfer payment and the degree of operational effort. And enterprise B needs to decide to build a platform, and one of the prerequisites for enterprise B to accept enterprise A's commission is that enterprise B needs to obtain non-negative income. To maximize $\pi_{B} (\theta )$, We have the derivative of $\pi_{B} (\theta )$: $\frac{{d\pi_{B} (\theta )}}{d\theta } = - k_{3} \theta < 0$. And, $\theta^{*} = \frac{p}{{(1 + \eta )k_{1} }}$, put $\theta^{*}$ into $\pi_{B} (\theta )$,$\pi_{B} (\theta^{*} ) = w_{1} - \frac{{k_{3} p^{2} }}{{2(1 + \eta )^{2} k_{1}^{2} }}$ Since $\pi_{B} (\theta^{*} ) \ge 0$, there is $w_{1} \ge \frac{{k_{3} p^{2} }}{{2(1 + \eta )^{2} k_{1}^{2} }}$. Put $\theta^{*}$ into $\pi_{A} (w_{1} ,e)$, we have $\pi_{A} (w_{1} ,e) = \frac{{p^{2} }}{{k_{1} }} + \alpha ep - w_{1} - \frac{{k_{2} e^{2} }}{2}$ To maximize $\pi_{A} (w_{1} ,e)$, we have the partial derivation of $\pi_{A} (w_{1} ,e)$:

$$ \left\{ {\begin{array}{*{20}c} {\frac{{\partial \pi_{A} (w_{1} ,e)}}{{\partial w_{1} }} = - 1 < 0} \\ {\frac{{\partial \pi_{A} (w_{1} ,e)}}{\partial e} = \alpha p - k_{2} e = 0} \\ \end{array} } \right. $$

And the result is

$$ \left\{ {\begin{array}{*{20}c} {w_{1}^{*} = \min \left( {\underline{{w_{1} }} ,\frac{{k_{3} p^{2} }}{{2(1 + \eta )^{2} k_{1}^{2} }}} \right)} \\ {e^{*} = \frac{\alpha p}{{k_{2} }}} \\ \end{array} } \right. $$

We discuss the results in three situations.

1. when $\frac{{k_{3} p^{2} }}{{2(1 + \eta )^{2} k_{1}^{2} }} \le \underline{{w_{1} }}$, $w_{1}^{*} = \underline{{w_{1} }}$, we obtain $\pi_{A} (w_{1}^{*} ,e^{*} ) = \frac{{p^{2} }}{{k_{1} }} + \frac{{\alpha^{2} p^{2} }}{{2k_{2} }} - \underline{{w_{1} }}$;
2. when $\underline{{w_{1} }} \le \frac{{k_{3} p^{2} }}{{2(1 + \eta )^{2} k_{1}^{2} }} < \overline{{w_{1} }}$, $w_{1}^{*} = \frac{{k_{3} p^{2} }}{{2(1 + \eta )^{2} k_{1}^{2} }}$, we get $\pi_{A} (w_{1}^{*} ,e^{*} ) = \frac{{p^{2} }}{{k_{1} }} + \frac{{\alpha^{2} p^{2} }}{{2k_{2} }} - \frac{{k_{3} p^{2} }}{{2(1 + \eta )^{2} k_{1}^{2} }}$;
3. if $\overline{{w_{1} }} \le \frac{{k_{3} p^{2} }}{{2(1 + \eta )^{2} k_{1}^{2} }}$, since $\pi_{B} (\theta^{*} ) < 0$, no solution.

### SBCO, mode3

When a railway service company chooses to build its own supply chain online platform and entrusts a third-party company to operate it, as in mode1, A needs to decide how hard it is to build the platform $\theta$, since platform operation is entrusted to a third-party company, A needs to make a decision to pay B's operating expenses $w_{{2}}$($\underline{{w_{{2}} }} \le w_{{2}} < \overline{{w_{{2}} }}$), and B needs to decide its operating effort $e$(enterprise A stipulates that the effect of enterprise B’s operation platform should be no worse than the effect of A’s self-operation, that is $e \ge \alpha^{2} p/\beta k_{2}$), the cost is $k_{{4}} e^{2} /2$($k_{{4}} < k_{{2}}$) accordingly. B has the corresponding operating experience which can bring more drainage effects, therefore, the drainage effect under the entrusted third-party enterprise operation model is $\beta e$($\beta > \alpha$). At this time, the profit function of the firm can be expressed as

$$ \begin{gathered} \begin{array}{*{20}c} {\max } & {\mathop {\pi_{A} }\limits_{{\underline{{w_{{2}} }} \le w_{{2}} < \overline{{w_{{2}} }} }} } \\ \end{array} (\theta ,w_{{2}} ) = (\theta + \beta e)p - \frac{{k_{1} \theta^{2} }}{{2}} - w_{2} \hfill \\ \begin{array}{*{20}l} {s.t.} \hfill & {\mathop {\pi_{B} }\limits_{{e \, \ge \, \frac{{\alpha^{2} p}}{{\beta k_{2} }}}} (e) = w_{2} - \frac{{k_{4} e^{2} }}{2}} \hfill \\ {} \hfill & {\pi_{B} (e^{*} ) \ge 0} \hfill \\ \end{array} \hfill \\ \end{gathered} $$

In the objective function above, the first term is total revenue, the second one is the cost of building the platform, and the third is the transfer payment for entrusting a third-party enterprise to operate on its behalf. Enterprise A needs to decide the degree of effort to build the platform and the size of transfer payment. And enterprise B needs to decide the degree of effort in operation, and one of the prerequisites for enterprise B to accept enterprise A's commission is that enterprise B needs to obtain non-negative income. To maximize $\pi_{B} (e)$, we have the derivation of $\pi_{B} (e)$,$\frac{{d\pi_{B} (e)}}{de} = - k_{4} e < 0$, then $e^{*} = \frac{{\alpha^{2} p}}{{\beta k_{2} }}$, put $e^{*}$ into $\pi_{B} (e)$, $\pi_{B} (e^{*} ) = w_{2} - \frac{{k_{4} \alpha^{4} p^{2} }}{{2\beta^{2} k_{2}^{2} }}$. Since $\pi_{B} (e^{*} ) \ge 0$, $w_{2} \ge \frac{{k_{4} \alpha^{4} p^{2} }}{{2\beta^{2} k_{2}^{2} }}$, put $e^{*}$ into $\pi_{A} (\theta ,w_{{2}} )$, we get $\pi_{A} (\theta ,w_{{2}} ) = \theta p + \frac{{\alpha^{2} p^{2} }}{{k_{2} }} - \frac{{k_{1} \theta^{2} }}{{2}} - w_{2}$. Maximize $\pi_{A} (\theta ,w_{{2}} )$, we have the partial derivation of $\pi_{A} (\theta ,w_{{2}} )$,

$$ \left\{ {\begin{array}{*{20}c} {\frac{{\partial \pi_{A} (\theta ,w_{{2}} )}}{\partial \theta } = p - k_{1} \theta = 0} \\ {\frac{{\partial \pi_{A} (\theta ,w_{{2}} )}}{{\partial w_{{2}} }} = - 1 < 0} \\ \end{array} } \right. $$

Then

$$ \left\{ {\begin{array}{*{20}c} {\theta^{*} = \frac{p}{{k_{{1}} }}} \\ {w_{{2}}^{*} = \min \left( {\underline{{w_{{2}} }} ,\frac{{k_{4} \alpha^{4} p^{2} }}{{2\beta^{2} k_{2}^{2} }}} \right)} \\ \end{array} } \right. $$

There are also three situations in it,

1. when $$\frac{{k_{4} \alpha^{4} p^{2} }}{{2\beta^{2} k_{2}^{2} }} \le \underline{{w_{{2}} }}$$, $$w_{{2}}^{*} = \underline{{w_{{2}} }}$$, $$\pi_{A} (\theta^{*} ,w_{{2}}^{*} ) = \frac{{p^{2} }}{{{2}k_{1} }} + \frac{{\alpha^{2} p^{2} }}{{k_{2} }} - \underline{{w_{{2}} }}$$;
2. when $$\underline{{w_{{2}} }} \le \frac{{k_{4} \alpha^{4} p^{2} }}{{2\beta^{2} k_{2}^{2} }} < \overline{{w_{{2}} }}$$, $$w_{{2}}^{*} = \frac{{k_{4} \alpha^{4} p^{2} }}{{2\beta^{2} k_{2}^{2} }}$$, $$\pi_{A} (\theta^{*} ,w_{{2}}^{*} ) = \frac{{p^{2} }}{{{2}k_{1} }} + \frac{{\alpha^{2} p^{2} }}{{k_{2} }} - \frac{{k_{4} \alpha^{4} p^{2} }}{{2\beta^{2} k_{2}^{2} }}$$;
3. when $\overline{{w_{{2}} }} \le \frac{{k_{4} \alpha^{4} p^{2} }}{{2\beta^{2} k_{2}^{2} }}$, since $\pi_{B} (e^{*} ) < 0$, thus, there is no solution.

### CCCO, mode4

When the railway service company chooses to outsource all the work, A only needs to decide how much to pay to B, the amount is represented with $w$($\underline{w} \le w < \overline{w}$), and B needs to decide the efforts degree $\theta$ of platform construction and the platform operation $e$. The profit revenue function is expressed as

$$ \begin{gathered} \begin{array}{*{20}c} {\max } & {\mathop {\pi_{A} }\limits_{{\underline{w} \le w < \overline{w} }} } \\ \end{array} (w) = [(1 + \eta )\theta + \beta e]p - w \hfill \\ \begin{array}{*{20}l} {s.t.} \hfill & {\mathop {\pi_{B} }\limits_{\begin{subarray}{l} \theta \, \ge \, \frac{p}{{(1 + \eta )k_{1} }} \\ e \, \ge \, \frac{{\alpha^{2} p}}{{\beta k_{2} }} \end{subarray} } (\theta ,e) = w - \frac{{k_{{3}} \theta^{2} }}{{2}} - \frac{{k_{4} e^{2} }}{{2}}} \hfill \\ {} \hfill & {\pi_{B} (\theta^{*} ,e^{*} ) \ge 0} \hfill \\ \end{array} \hfill \\ \end{gathered} $$

Since A entrusts platform construction and operation to enterprise B, the above objective function contains only two items. The first item is the total revenue, and the second item is the transfer payment for entrusting a third-party enterprise to build the platform and operate it. Enterprise A needs to decide the size of the transfer payment. And enterprise B needs the effort of decision-making platform construction and operation effort, and one of the prerequisites for enterprise B to accept the commission of enterprise A is that enterprise B needs to obtain non-negative income. To maximize $\pi_{B} (\theta ,e)$, we need to have a partial derivation of $\pi_{B} (\theta ,e)$ and let it equal 0,

$$ \left\{ {\begin{array}{*{20}c} {\frac{{\partial \pi_{B} (\theta ,e)}}{\partial \theta } = - k_{3} \theta < 0} \\ {\frac{{\partial \pi_{B} (\theta ,e)}}{\partial e} = - k_{4} e < 0} \\ \end{array} } \right. $$

Then

$$ \left\{ {\begin{array}{*{20}c} {\theta^{*} = \frac{p}{{(1 + \eta )k_{1} }}} \\ {e^{*} = \frac{{\alpha^{2} p}}{{\beta k_{2} }}} \\ \end{array} } \right. $$

Put $(\theta^{*} ,e^{*} )$ into $\pi_{B} (\theta ,e)$, then $\pi_{B} (\theta^{*} ,e^{*} ) = w - \frac{{k_{3} p^{2} }}{{2(1 + \eta )^{2} k_{1}^{2} }} - \frac{{k_{4} \alpha^{4} p^{2} }}{{2\beta^{2} k_{2}^{2} }}$. Since $\pi_{B} (\theta^{*} ,e^{*} ) \ge 0$, therefore, $w \ge \frac{{k_{3} p^{2} }}{{2(1 + \eta )^{2} k_{1}^{2} }} + \frac{{k_{4} \alpha^{4} p^{2} }}{{2\beta^{2} k_{2}^{2} }}$;Put $(\theta^{*} ,e^{*} )$ into $\pi_{A} (w)$,$\pi_{A} (w) = \frac{{p^{2} }}{{k_{{1}} }}{ + }\frac{{\alpha^{{2}} p^{2} }}{{k_{{2}} }} - w$; Maximize $\pi_{A} (w)$, we have the derivation of $\pi_{A} (w)$, get $\frac{{d\pi_{A} (w)}}{dw} = - 1 < 0$; $w^{*} = \min \left( {\underline{w} ,\frac{{k_{3} p^{2} }}{{2(1 + \eta )^{2} k_{1}^{2} }} + \frac{{k_{4} \alpha^{4} p^{2} }}{{2\beta^{2} k_{2}^{2} }}} \right)$.

There are 3 situations:

1. when $\frac{{k_{3} p^{2} }}{{2(1 + \eta )^{2} k_{1}^{2} }} + \frac{{k_{4} \alpha^{4} p^{2} }}{{2\beta^{2} k_{2}^{2} }} \le \underline{w}$, $w^{*} = \underline{w}$, we obtain $\pi_{A} (w^{*} ) =\frac{{p^{2} }}{{k_{1} }} + \frac{{\alpha^{2} p^{2} }}{{k_{2} }} - \underline{w}$;
2. when $\underline{w} \le \frac{{k_{3} p^{2} }}{{2(1 + \eta )^{2} k_{1}^{2} }} + \frac{{k_{4} \alpha^{4} p^{2} }}{{2\beta^{2} k_{2}^{2} }} < \overline{w}$, $w^{*} = \frac{{k_{3} p^{2} }}{{2(1 + \eta )^{2} k_{1}^{2} }} + \frac{{k_{4} \alpha^{4} p^{2} }}{{2\beta^{2} k_{2}^{2} }}$; we get $\pi_{A} (w^{*} ) =\frac{{p^{2} }}{{k_{1} }} + \frac{{\alpha^{2} p^{2} }}{{k_{2} }} - \frac{{k_{3} p^{2} }}{{2(1 + \eta )^{2} k_{1}^{2} }} - \frac{{k_{4} \alpha^{4} p^{2} }}{{2\beta^{2} k_{2}^{2} }}$;
3. $\overline{w} \le \frac{{k_{3} p^{2} }}{{2(1 + \eta )^{2} k_{1}^{2} }} + \frac{{k_{4} \alpha^{4} p^{2} }}{{2\beta^{2} k_{2}^{2} }}$, since $\pi_{B} (\theta^{*} ,e^{*} ) < 0$, there is no solution.

## Analysis of the four modes

The four modes are modeled and solved above. Now we compare the four modes and draws corresponding conclusions.

## Mode 1 vs mode 2

**Corollary 1**: when $\underline{{w_{1} }} \ge \frac{{p^{2} }}{{2k_{1} }}$ or $\overline{{w_{1} }} \le \frac{{k_{3} p^{2} }}{{2(1 + \eta )^{2} k_{1}^{2} }}$, Mode1gives priority to Mode2; or else, Mode2 is better than Mode1.

Under Mode 1, the profit of enterprise A is:$\pi_{A} (\theta^{*} ,e^{*} ) = \frac{{p^{2} }}{{2k_{1} }} + \frac{{\alpha^{2} p^{2} }}{{2k_{2} }}$; In Mode2,

$$ \pi_{A} (w_{1}^{*} ,e^{*} ) = \left\{ {\begin{array}{*{20}l} {\frac{{p^{2} }}{{k_{1} }} + \frac{{\alpha^{2} p^{2} }}{{2k_{2} }} - \underline{{w_{1} }} ,} \hfill & {\frac{{k_{3} p^{2} }}{{2(1 + \eta )^{2} k_{1}^{2} }} \le \underline{{w_{1} }} } \hfill \\ {\frac{{p^{2} }}{{k_{1} }} + \frac{{\alpha^{2} p^{2} }}{{2k_{2} }} - \frac{{k_{3} p^{2} }}{{2(1 + \eta )^{2} k_{1}^{2} }},} \hfill & {\underline{{w_{1} }} \le \frac{{k_{3} p^{2} }}{{2(1 + \eta )^{2} k_{1}^{2} }} < \overline{{w_{1} }} } \hfill \\ {\begin{array}{*{20}c} {{\text{No}}} & {{\text{value}}} \\ \end{array} ,} \hfill & {\overline{{w_{1} }} \le \frac{{k_{3} p^{2} }}{{2(1 + \eta )^{2} k_{1}^{2} }}} \hfill \\ \end{array} } \right. $$

While $\frac{{k_{3} p^{2} }}{{2(1 + \eta )^{2} k_{1}^{2} }} \le \underline{{w_{1} }}$, $\pi_{A} (\theta^{*} ,e^{*} ) - \pi_{A} (w_{1}^{*} ,e^{*} ) = \underline{{w_{1} }} - \tfrac{{p^{2} }}{{2k_{1} }}$, then $\underline{{w_{1} }} < \frac{{p^{2} }}{{2k_{1} }}$, Mode2 is better than Mode1; and when $\underline{{w_{1} }} \ge \frac{{p^{2} }}{{2k_{1} }}$, Mode1 is better than Mode2. When $\underline{{w_{1} }} \le \frac{{k_{3} p^{2} }}{{2(1 + \eta )^{2} k_{1}^{2} }} < \overline{{w_{1} }}$, $\pi_{A} (\theta^{*} ,e^{*} ) - \pi_{A} (w_{1}^{*} ,e^{*} ) = \frac{{p^{2} }}{{2k_{1} }}[\frac{{k_{3} }}{{(1 + \eta )^{2} k_{1} }} - 1] < 0$, and Mode2 is better than Mode1. Whereas $\overline{{w_{1} }} \le \frac{{k_{3} p^{2} }}{{2(1 + \eta )^{2} k_{1}^{2} }}$, there is no value in Mode2, thus only one option for Mode1. And because of $k_{3} < k_{1}$, then $\frac{{k_{3} p^{2} }}{{2(1 + \eta )^{2} k_{1}^{2} }} < \frac{{p^{2} }}{{2k_{1} }}$ is constantly true, therefore, Corollary1 is approved.

The completion 1 shows that when firm A gives a large lower bound or a small upper bound on the platform construction budget, firm A will choose to build and operate its own platform rather than delegate the construction and operation of the platform. Specifically, when the lower limit of the platform construction budget given by firm A is large, firm B will only meet A's minimum requirements in terms of platform construction, but will receive a very high transfer payment from A, which is uneconomical for A. When the upper limit of the platform construction budget given by firm A is small, firm B will undertake the platform construction work and will not receive positive revenue, and for this reason, firm B is not likely to undertake the platform construction work.

### Mode 1 vs mode 3

**Corollary 2**: When $\underline{{w_{{2}} }} \ge \frac{{\alpha^{{2}} p^{2} }}{{2k_{{2}} }}$ or $\overline{{w_{{2}} }} \le \frac{{k_{{4}} \alpha^{{4}} p^{2} }}{{2\beta^{2} k_{{2}}^{2} }}$, Mode1 is better than Mode3, otherwise, Mode3 is better.

Proof is sketchy because it is the same process as to prove Corollary 1.

Corollary 2 shows that when firm A gives a very large lower or very small upper bound on its operating budget, firm A will choose to build and operate its own operations rather than build and commission its own operations. Specifically, when firm A gives a large lower bound on its operating budget, firm B will only meet A's minimum requirements in terms of operations but receives a very high transfer payment from A, which is uneconomical for A. When firm A gives a very small upper bound on its operating budget, firm B will take over operations and will not receive a positive return, and for this reason, firm B will not be able to take over operations.

### Mode 1 vs mode 4

**Corollary 3**: when $\underline{w} \ge \frac{{p^{2} }}{{2k_{{1}} }}{ + }\frac{{\alpha^{{2}} p^{2} }}{{2k_{{2}} }}$ or $\overline{w} \le \frac{{k_{3} p^{2} }}{{2(1 + \eta )^{2} k_{1}^{2} }} + \frac{{k_{{4}} \alpha^{{4}} p^{2} }}{{2\beta^{2} k_{{2}}^{2} }}$, Mode1 is better, otherwise, is Mode4.

Proof is slightly, and the same reason of proof process.

Corollary3 shows that when enterprise A gives a large lower or small upper budget for platform construction and operation, enterprise A will choose to build and operate its own platform instead of commissioning construction and operation. Specifically, when the lower limit of the platform construction and operation budget given by firm A is large, firm B will only meet A's minimum requirements in terms of platform construction and operation, but receive a high transfer payment from A, which is uneconomical for A. When the upper limit of the platform construction and operation budget given by firm A is small, firm B will undertake the platform construction and operation work, but will not receive positive revenue. May undertake the construction and operation of the platform.

## Numerical simulation

The four modes involve a large number of parameters, and it is very complicated to analyze them simultaneously.

Considered the reality, we select parameters as follow: $k_{1} = 10$, $k_{{2}} = {2}0$, $k_{{3}} = {8}$, $k_{{4}} = {15}$, $\eta { = 0}{\text{.1}}$, $\underline{{w_{1} }} = 1$, $\overline{{w_{1} }} = 10$, $\underline{{w_{{2}} }} = 1$, $\overline{{w_{{2}} }} = 10$, $\underline{w} = {2}$ and $\overline{w} = 1{8}$. In addition, in terms of the fact that the operating capabilities of the partners and the nature of the platform's products have a greater impact on the final selection of the four models, we develop numerical examples to explore them in this section.

**Fig. 3:**
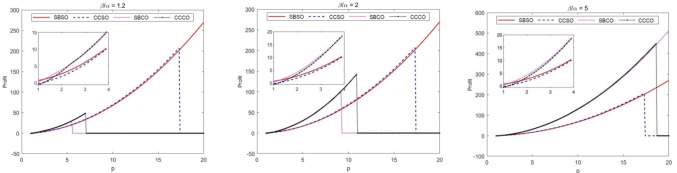
Analysis of the impact of the partner's operating capabilities

From Fig. 3, it can be seen that the optimal model is influenced by the nature of the platform operating product and the operating capacity of the partner. Figure 1(1) shows that when the operating capability of the partner is only slightly better than that of the railway service enterprise, Mode1 is optimal if the revenue per passenger brought by the platform operating product is very low; if the revenue per passenger brought by the platform operating product is low, the effect of entrusted operation is better than self-operated operation; if the revenue per passenger brought by the platform operating product is high, Mode 2 is optimal; if the revenue per passenger brought by the platform operating product is low, the effect of entrusted operation is better than self-operated operation; if the revenue per passenger brought by the platform operating product is high, the effect of entrusted operation is better than self-operated operation. The revenue per customer reaches a very high level and Mode 1 is again the optimal choice. The reason for the above conclusion is that there is no cost budget constraint for self-build and self-operate, while the other three models are constrained by the upper and lower limits of cost budget. To enable rail service companies to achieve higher returns in the same situation, it is suggested that rail service companies can increase their commissioning budgets moderately. The conclusion of Fig. 1(2) is almost identical to Fig. 1(1) in that the revenue curves of mode 1 and mode 2 are identical, and the non-zero revenue curves of mode 3 and mode 4 are moderately extended. This implies that all other things being equal, the advantage of delegated operation becomes more pronounced as the operating capacity of the partner increases. Figure 1(3) confirms this point even more, and the comparison of the vertical coordinates between Fig. 1(3) and the two aforementioned sub-figures shows that all other conditions being equal, finding a partner with greater operational capacity can bring double the profit for the railway service enterprise.

In reality, a railway service company needs to decide on the range of products or services to be operated on the platform. For example, a railway service enterprise can provide only basic railway services including housekeeping and maintenance, or it can provide high-end railway services including study and tutoring, finance, etc. This sub-section examines the issue from the perspective of how exactly a railway service enterprise should ring-fence its scope of services.

**Fig. 4:**
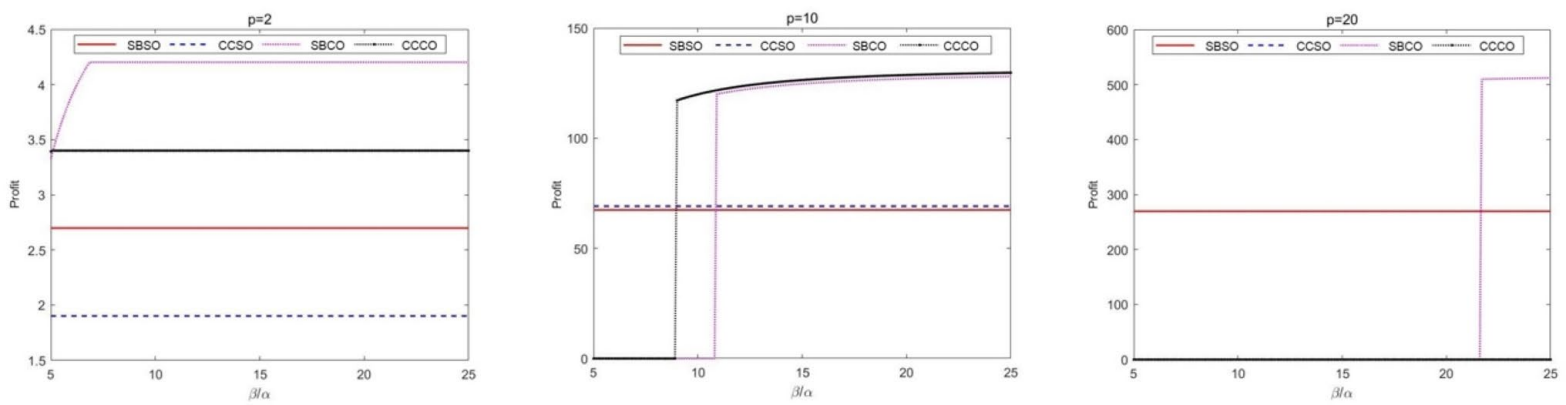
Analysis of the impact of the nature of platform management products

There are three subgraphs in Fig. 4, and subgraph 2 (1) shows that when the product or service operated by the railway service enterprise's platform is more basic, the commissioning is better, and the optimal model changes from Mode 4 to Mode 3 as the partner's operational capacity increases. It should be noted that when the products or services operated by the platform are more basic, the railway service enterprise gains little. When the products or services operated by the platform of the railway service enterprise are moderate, entrusted self-operation is always better than self-built self-operation, and when the operating capacity of the partner is weak, the railway service enterprise will choose self-operation, and with the enhancement of the operating capacity of the partner, the revenue brought by entrusted operation will be better than self-operation, and entrusted entrustment is always better than self-built entrustment. When the products or services operated by the railway service enterprise platform are more advanced, the railway service enterprise will always choose mode one unless the partner's operational capability is superior.

## Findings

Furthermore, the optimal strategy is influenced by the nature of the platform operation product and the operating capacity of the partner. When the operating capability of the partner is only a little better than that of the railway service enterprise, Mode 1 is optimal if the revenue per passenger brought by the platform operating product is very low; if the revenue per passenger brought by the platform operating product is low, the effect of entrusted operation is better than self-operation; if the revenue per passenger brought by the platform operating product is high, Mode 2 is optimal; once the revenue per passenger brought by the platform operating product reaches very low, the effect of entrusted operation is better than self-operation. At a high level, Model 1 is once again the optimal choice. And finding a partner with strong operational capability may be able to bring double the profits. From another point of view, when the products or services operated by the platform of railway service enterprises are relatively basic, the effect of entrusted operation is better, and as the operating capacity of the partner increases, the optimal mode changes from Mode 4 to Mode 3; when the products or services operated by the platform of railway service enterprises are moderate, entrusted self-operation is always better than self-constructed self-operation, and when the operating capacity of the partner is weak, railway service enterprises will choose self-operated operation. operations, as the partner's operational capability increases, the revenue generated by commissioning will be better than self-operation, and commissioning will always be better than self-constructed commissioning; when the products or services operated by the railway service company's platform are more advanced, the railway service company will always choose mode one unless the partner's operational capability is superior.

Establishing a platform successfully is the basis for railway service companies to meet the demands of O2O supply chain services, therefore, railway service companies need to transform the traditional multi-level management model into a flat model which aims to ensure the normal operation of the entire O2O supply chain service. According to the results, we find that when the lower budget limit given by the railway service enterprise is very large, the entrusted enterprise will only meet the minimum requirements of the railway service enterprise, but will receive a high transfer payment from the railway service enterprise, and the railway service enterprise will choose to build/operate itself; when the upper budget limit given by the railway service enterprise is very small, the entrusted enterprise refuses to undertake the relevant work because it will not receive positive income, and the railway service enterprise will choose to build/operate itself. Rail service companies can only choose to build/operate on their own. In other cases, delegated build/operate works better for the railway service enterprise. In this paper, the proposed model only considers the cost of O2O platform construction. However, the influencing factors of O2O platform establishment are diverse, in which the railway passage flow volume are important other than cost. Therefore, number of visitors may be considered as a factor to the model to help railway enterprises to make decision.
